# Supplementary material for: An Eco-Friendly Method to Get a Bio-Based Dicarboxylic Acid Monomer 2,5-Furandicarboxylic Acid and Its Application in the Synthesis of Poly(hexylene 2,5-furandicarboxylate) (PHF)
Source: Polymers (Basel). 2019 Jan 23;11(2):197. doi: 10.3390/polym11020197 (PMC6418886; doi:10.3390/polym11020197)
Supplement: Supplementary file 1 [file polymers-11-00197-s001.pdf]

Supporting Information

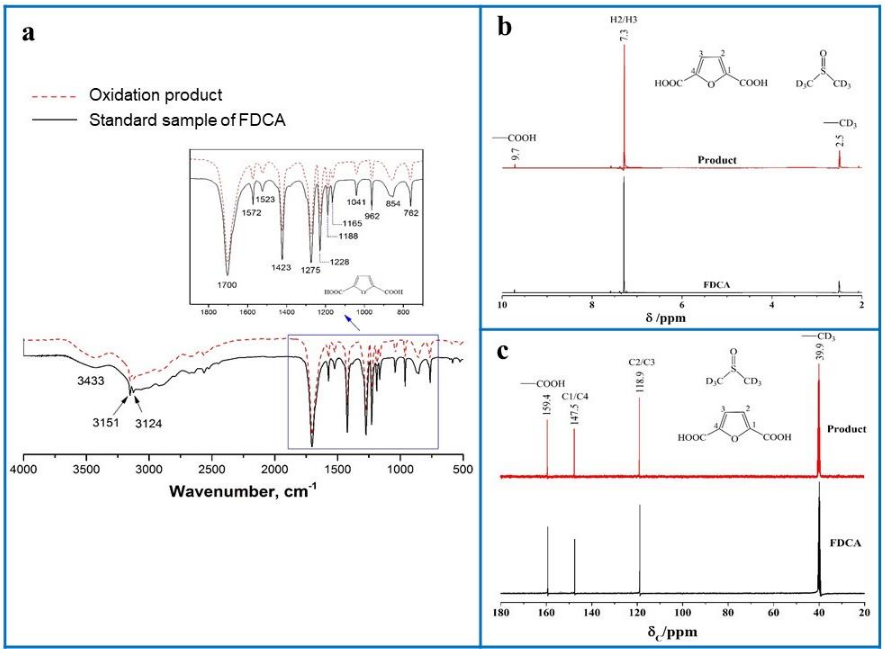

Figure S1. Typical FTIR and NMR spectrum of the oxidation product and FDCA standard sample (a FTIR, b ^{1}H NMR, c ^{13}C NMR)
